# Individualized cortical gyrification in neonates with congenital heart disease

**DOI:** 10.1093/braincomms/fcae356

**Published:** 2024-10-07

**Authors:** Daniel Cromb, Siân Wilson, Alexandra F Bonthrone, Andrew Chew, Christopher Kelly, Manu Kumar, Paul Cawley, Ralica Dimitrova, Tomoki Arichi, J Donald Tournier, Kuberan Pushparajah, John Simpson, Mary Rutherford, Joseph V Hajnal, A David Edwards, Chiara Nosarti, Jonathan O’Muircheartaigh, Serena J Counsell

**Affiliations:** Centre for the Developing Brain, School of Biomedical Engineering and Imaging Sciences, King’s College London, London SE1 7EH, UK; Centre for the Developing Brain, School of Biomedical Engineering and Imaging Sciences, King’s College London, London SE1 7EH, UK; Fetal-Neonatal Neuroimaging and Developmental Science Center, Boston Children's Hospital, Boston, MA 02115, USA; Division of Newborn Medicine, Boston Children's Hospital, Boston, MA 02115, USA; Department of Pediatrics, Harvard Medical School, Boston, MA 02115, USA; Centre for the Developing Brain, School of Biomedical Engineering and Imaging Sciences, King’s College London, London SE1 7EH, UK; Centre for the Developing Brain, School of Biomedical Engineering and Imaging Sciences, King’s College London, London SE1 7EH, UK; Centre for the Developing Brain, School of Biomedical Engineering and Imaging Sciences, King’s College London, London SE1 7EH, UK; GKT Medical School, King’s College London, London SE1 7EH, UK; Centre for the Developing Brain, School of Biomedical Engineering and Imaging Sciences, King’s College London, London SE1 7EH, UK; MRC Centre for Neurodevelopmental Disorders, King's College London, London SE1 1UL, UK; Centre for the Developing Brain, School of Biomedical Engineering and Imaging Sciences, King’s College London, London SE1 7EH, UK; Centre for the Developing Brain, School of Biomedical Engineering and Imaging Sciences, King’s College London, London SE1 7EH, UK; MRC Centre for Neurodevelopmental Disorders, King's College London, London SE1 1UL, UK; Paediatric Neurosciences, Evelina London Children's Hospital, London SE1 7EH, UK; Centre for the Developing Brain, School of Biomedical Engineering and Imaging Sciences, King’s College London, London SE1 7EH, UK; Department of Cardiovascular Imaging, King’s College London, London SE1 7EH, UK; Department of Fetal and Paediatric Cardiology, Evelina London Children’s Hospital, London SE1 7EH, UK; Department of Cardiovascular Imaging, King’s College London, London SE1 7EH, UK; Department of Fetal and Paediatric Cardiology, Evelina London Children’s Hospital, London SE1 7EH, UK; Centre for the Developing Brain, School of Biomedical Engineering and Imaging Sciences, King’s College London, London SE1 7EH, UK; MRC Centre for Neurodevelopmental Disorders, King's College London, London SE1 1UL, UK; Centre for the Developing Brain, School of Biomedical Engineering and Imaging Sciences, King’s College London, London SE1 7EH, UK; Centre for the Developing Brain, School of Biomedical Engineering and Imaging Sciences, King’s College London, London SE1 7EH, UK; Centre for the Developing Brain, School of Biomedical Engineering and Imaging Sciences, King’s College London, London SE1 7EH, UK; Department of Child and Adolescent Psychiatry, Institute of Psychiatry, Psychology and Neuroscience, King’s College London, London SE5 8AB, UK; Centre for the Developing Brain, School of Biomedical Engineering and Imaging Sciences, King’s College London, London SE1 7EH, UK; Paediatric Neurosciences, Evelina London Children's Hospital, London SE1 7EH, UK; Department of Forensic and Neurodevelopmental Sciences, Institute of Psychiatry, Psychology and Neuroscience, King’s College London, London SE5 8AB, UK; Centre for the Developing Brain, School of Biomedical Engineering and Imaging Sciences, King’s College London, London SE1 7EH, UK

**Keywords:** cerebral oxygen delivery, MRI, neurodevelopmental outcomes

## Abstract

Congenital heart disease is associated with impaired early brain development and adverse neurodevelopmental outcomes. This study investigated how individualized measures of preoperative cortical gyrification index differ in 142 infants with congenital heart disease, using a normative modelling approach with reference data from 320 typically developing infants. Gyrification index *Z*-scores for the whole brain and six major cortical areas were generated using two different normative models: one accounting for post-menstrual age at scan, post-natal age at scan and sex, and another additionally accounting for supratentorial brain volume. These *Z*-scores were compared between congenital heart disease and control groups to test the hypothesis that cortical folding in infants with congenital heart disease deviates from the normal developmental trajectory. The relationships between whole-brain gyrification index *Z*-scores from the two normative models and both cerebral oxygen delivery and neurodevelopmental outcomes were also investigated. Global and regional brain gyrification was significantly reduced in neonates with congenital heart disease, but not when supratentorial brain volume was accounted for. This finding suggests that whilst cortical folding is reduced in congenital heart disease, it is primarily driven by a reduction in brain size. There was a significant positive correlation between cerebral oxygen delivery and whole-brain gyrification index *Z*-scores in congenital heart disease, but not when supratentorial brain volume was accounted for. Cerebral oxygen delivery is therefore likely to play a more important role in the biological processes underlying volumetric brain growth than cortical folding. No significant associations between whole-brain gyrification index *Z*-scores and motor/cognitive outcomes or autism traits were identified in the 70 infants with congenital heart disease who underwent neurodevelopmental assessment at 22-months. Our results suggest that chronic *in utero* and early post-natal hypoxia in congenital heart disease is associated with reductions in cortical folding that are proportional to reductions in supratentorial brain volume.

## Teaser

Cromb, Wilson *et al*. used normative modelling with reference data from 320 typically developing infants to assess individualized measures of cortical gyrification in 142 infants with congenital heart disease (CHD). They show that whilst cortical folding is reduced in CHD, this reduction is proportional to reductions in brain volume.

## Graphical Abstract

**Graphical Abstract:**
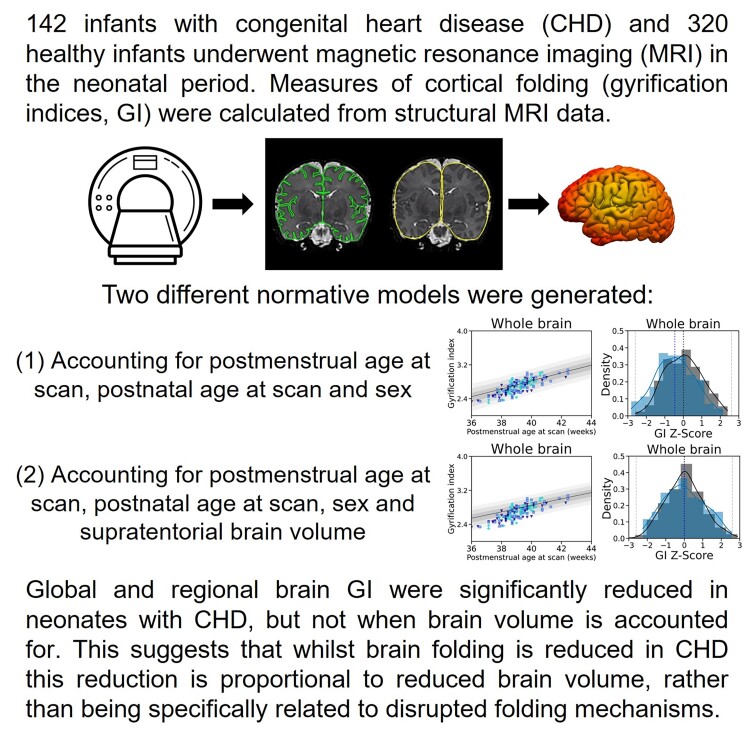

## Introduction

Cortical development is a complex process that begins *in utero* and continues into adulthood.^1-3^ The foundations for cortical architecture are laid during the foetal period through a programmed sequence of processes, including neurogenesis, neuronal migration and the formation of sulci and gyri.^4,5^ These fundamental processes determine the highly conserved pattern of primary cortical folds, and are critical for subsequent post-natal brain development.^6-8^ Cortical development in the neonatal period is characterized by further expansion of the sulci and deepening of the gyri, increasing cortical curvature and surface area.^6,9-11^ The extent of folding across the cerebral cortex can be represented by the gyrification index (GI), which serves as a marker of prenatal and early post-natal cortical development.^12^

Congenital heart disease (CHD) is the most common congenital malformation^13^ and is associated with altered early brain development,^14-19^ as well as an increased risk of neurodevelopmental impairments,^17,20,21^ which can persist into adulthood.^22-24^ Understanding the biological mechanisms of neurodevelopmental impairments in the CHD population remains an important challenge to resolve if outcomes are to be improved.

Previous studies have identified significantly altered cortical development in foetuses and infants with CHD including reduced GI of newborns with CHD in autopsy specimens^25^ and in both foetal^26,27^ and neonatal.^28,29^ MRI studies, as well as altered cortical microstructure.^30^ Previous work by our group revealed that reduced cortical GI in newborns with CHD is associated with impaired cerebral oxygen delivery (CDO_2_).^15^ However, this work was performed in a relatively small cohort using a traditional case-control comparison approach without accounting for individual variability across the population.

Normative modelling is a strategy for mapping individual data points from a reference distribution (usually derived from a control cohort) enabling the quantification of deviations from typical development for individual infants. This approach has been used previously to investigate aberrant volumetric growth in infants with CHD both prior to surgery^31^ and in the perioperative period.^32^ However, to date, normative modelling has not been used to study cortical gyrification in neonates with CHD. It is also an ideal tool for studying metrics such as cortical folding where individual variance is likely to be high.

### Study aims

The aim of this study was to test the hypothesis that preoperative cortical gyrification in infants with CHD deviates from the normal trajectory of development, as defined using a large cohort of healthy infants. We also explored whether altered preoperative cortical gyrification in infants with CHD was associated with impaired neurodevelopmental outcomes at 22 months.

## Materials and methods

### Ethical approval

The National Research Ethics Service West London committee provided ethical approval (CHD: 07/H0707/105 & 21/WA/0075; dHCP: 14/LO/1169). Informed written parental consent was obtained before the collection of MRI and neurodevelopmental assessment data.

### CHD participants

#### Recruitment and categorisation

Infants with critical or serious CHD, based on previously published classifications^33^ were recruited prospectively from the Neonatal and Paediatric Intensive Care Units at the Evelina London Children’s Hospital (Guy’s and St. Thomas’ NHS Trust), between 2015 and 2022. Critical CHD included infants with hypoplastic left heart syndrome (HLHS), interrupted aortic arch (IAA), pulmonary atresia (PA) with an intact ventricular septum, simple transposition of the great arteries (TGA) and all infants requiring surgery or cardiac catheterisation within the first 28 days after birth with any of the following: aortic valve stenosis (AS), coarctation of the aorta (CoA), pulmonary valve stenosis (PS), PA with ventricular septal defect, tetralogy of Fallot (ToF) and total anomalous pulmonary venous drainage (TAPVD). Serious CHD was defined as any cardiac lesion not defined as critical, requiring cardiac catheterisation or surgery between 1 month and 1 year of age. Each infant was also allocated to one of three diagnostic CHD categories based on the haemodynamic impact of the underlying cardiac diagnosis, using the sequential segmental approach^34^: (i) abnormal streaming of blood; (ii) left-sided heart lesions; or (iii) right-sided heart lesions.

#### Inclusion and exclusion criteria

Infants with CHD were eligible for inclusion in this analysis if they did not undergo any neonatal surgery prior to the MRI, and if preoperative imaging was performed between 36 and 46 weeks post-menstrual age (PMA).

### Typically developing participants

To model typical development, we used the open-access developing Human Connectome Project (dHCP; http://developingconnectome.org/) data as our reference cohort, available at: https://nda.nih.gov/edit_collection.html?id=3955. Infants were included if they were born after 37 weeks, scanned between 37 and 45 weeks PMA, and if no major brain lesions (e.g. cortical infarcts) were reported on their neuroimaging.

### Magnetic resonance imaging

MRI for CHD infants and healthy participants was performed on a Philips Achieva 3 tesla system (Best, NL) situated on the Neonatal Unit at St Thomas’ Hospital, London. Imaging was performed during natural sleep without sedation. Pulse oximetry, respiratory rate, temperature and electrocardiography were monitored throughout by a nurse and/or paediatrician experienced in neonatal MRI procedures and resuscitation. Data were acquired with a dedicated 32-channel receive neonatal head coil and positioning system (Rapid Biomedical GmbH, Rimpar DE).^35^ Scans included a 5-s noise ramp-up to avoid a startle response. T2-weighted multi-slice turbo spin echo scans were acquired in two stacks in sagittal and axial planes [repetition time (TR)/echo time (TE) = 12 000/156 ms; flip angle = 90°; slice thickness = 1.6 mm; slice overlap = 0.8 mm; in-plane resolution: 0.8 × 0.8 mm; SENSE factor = 2.11/2.58 (axial/sagittal)]. Diffusion-weighted MRI, susceptibility-weighted MRI, and venography were also acquired, as previously reported,^16^ but not utilized in this study.

#### Cerebral blood flow (CBF) and CDO_2_ in CHD infants

For CHD infants, quantitative flow imaging was also performed using velocity sensitized phase contrast imaging. A single-slice T1-weighted fast-field-echo sequence was acquired in a plane perpendicular to both internal carotid and basilar arteries, at the level of the sphenoid bone,^36^ as reported by Kelly *et al*.^15^ Scan parameters were: field of view: 100 × 100 mm^2^, acquisition resolution: 0.6 × 0.6 × 4.0 mm^2^, TR: 6.4 ms, TE: 4.3 ms, flip angle: 10°, 20 repetitions, maximal encoding velocity: 140 cm s^−1^, scan time: 71 s. Regions of interest (ROIs) were drawn manually around the three vessels, using Segment v2.0 R480045,^37^ and flow curves generated. An estimate of total CBF was calculated from the sum of these vessels.^38^ Haemoglobin (Hb) levels were measured as part of routine clinical care in all CHD participants prior to the scan. Pre-ductal arterial oxygen saturation (SaO_2_) was measured at the time of scan using a pulse-oximeter applied to the right hand. CDO_2_ was calculated using the following formulae^39^:


$$\mathrm{CD}O_{2}(\mathrm{ml}\;O_{2}\;\mathrm{mi}n^{-1})=\mathrm{Sa}O_{2}x\;[\mathrm{Hb}](g\;dl^{-1})x\;1.36\;x\;[\mathrm{CBF}](\mathrm{ml}\;\mathrm{mi}n^{-1}),$$

where 1.36 is the amount of oxygen bound per gram of Hb at one atmosphere (Hüfner’s constant).^40^

CBF data were obtained in 120 of the 142 infants with CHD [mean (Sd) CBF = 85.2 ± 20.2 ml min^−1^]. Hb levels [mean (Sd) = 167 ± 24 g dl^−1^] were measured as part of routine clinical care in infants with CHD at a median of 2 days (range 1–4 days) prior to the scan.

### Brain region and tissue segmentation

T2-weighted volumes were reconstructed using a dedicated algorithm to correct motion and integrate data from both acquired stacks (reconstructed voxel size = 0.5 mm^3^).^41,42^ The motion-corrected T2-weighted images underwent bias field correction and brain extraction before being segmented into eight tissue classes: cortical grey matter (cGM), white matter, total deep grey matter, cerebellum, brainstem, hippocampus and amygdala, ventricles and extracerebral CSF and brain regions using an automated, validated, neonatal-specific processing pipeline developed for the dHCP study.^43-45^ These segmentations were then used to generate cortical, white matter and pial surfaces. All surface reconstructions that completed successfully were visually reviewed and those where the surface processing pipeline had failed to generate anatomically accurate surfaces were excluded from further analysis (*N* = 27; 16 control, 11 CHD). Supratentorial brain volume (STBV) was calculated by summing cGM, white matter, deep grey matter and hippocampus and amygdala volumes.

### GI calculation

**Figure 1:**
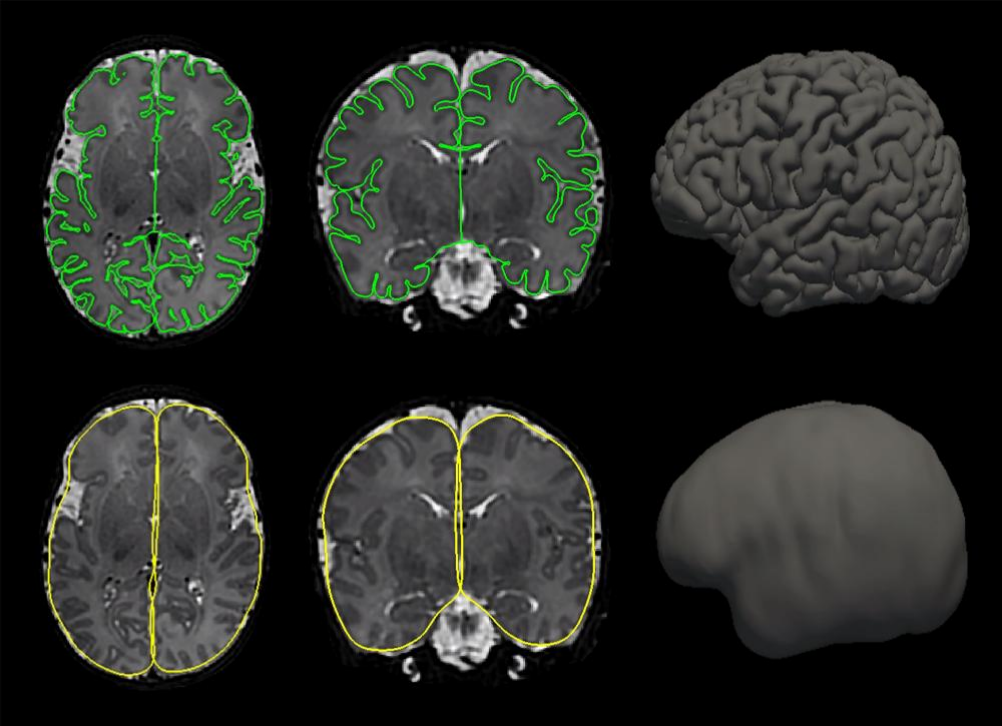
**Cortical surfaces.** Examples of the left and right hemisphere pial surfaces (green) and outer smoothed surfaces (yellow) used for calculation of the GI, overlaid on T2-weighted brain MRIs for one subject, acquired at 39^+3^ weeks. The surface renderings of both the pial and outer smoothed surfaces are shown alongside.

Pial surfaces generated by the dHCP pipeline were converted to FreeSurfer compatible formats using the ‘compute_lgi’ command in FreeSurfer^46^ (https://surfer.nmr.mgh.harvard.edu/fswiki/LGI), and an outer smoothed surface which tightly wraps the pial surface was generated. Examples of the derived left and right hemisphere pial and smoothed outer surfaces used for calculation of the GI, overlaid on the subject’s T2-weighted image are shown in Fig. 1. Following this, a local GI-value was calculated for every vertex on the surface of the brain. This method for quantifying cortical folding inspired by Zilles and Armstrong,^12,47^ compares the ratio between the area of two circular ROIs overlayed on the pial and outer smoothed surfaces, effectively measuring the amount of cortex buried within the sulcal folds as compared with the amount of visible cortex in local circular ROIs.^48,49^

To calculate total brain GI, we took the average value of all the individual vertex GI-values. GI-values for all cortical regions in the drawEM atlas^44^ corresponding to the frontal, occipital, parietal, temporal, cingulate and insular cortices were averaged to calculate GI measurements for each of these regions.

### MRI review

All MRIs were reported by experienced perinatal neuroradiologists. The presence of white matter injury, arterial ischaemic stroke, cerebellar haemorrhage, cerebral sinus venous thrombosis, intraparenchymal haemorrhage and ventriculomegaly were recorded. Infants with CHD were excluded from analysis if they had arterial ischaemic strokes affecting the cortex on MRI, as these prevent accurate segmentation and reconstruction of the cortical ribbon.

### Modelling typical cortical gyrification

Typical brain cortical gyrification was modelled using MR data acquired for the dHCP for infants with cognitive and motor outcomes within two Sd of the normative mean at 18 months (as assessed using Bayley-III scales of infant and toddler neurodevelopment^50^), using Gaussian Process Regression (GPR), or normative modelling, as described by Kelly *et al*. and Dimitrova^31,51^ (https://github.com/ralidimitrova/GPR_NeoVols). Briefly, GPR is a Bayesian non-parametric regression method which can be used to model trajectories of metrics of typical brain development between specified gestational ages. Here, GPR was used to generate separate normative trajectories for total brain GI, as well as GI for the frontal, occipital, parietal, temporal, cingulate and insular cortices.

These GPR models were then used to assess deviation from the normative growth trajectory for each individual infant with CHD. The difference between predicted and observed values, normalized by the predictive confidence, represents the deviation of a measurement from the expected mean. Two separate normative models were generated: Model 1 that accounted for the infant’s PMA at scan, post-natal age at scan (i.e. the number of days since birth), and sex, and Model 2 that accounted for STBV in addition to PMA at scan, post-natal age at scan and sex. For each of these models, a *Z*-score quantifying the degree of atypicality for cortical gyrification for each baby with CHD was calculated. Fitting these two different models enables an exploration of how changes in GI *Z*-scores are influenced by STBV.

### Neurodevelopmental outcomes

Infants with CHD were invited to attend a follow-up neurodevelopmental assessment at 22-months of age. Infants completed the Bayley Scales of Infant and Toddler Development–Third Edition (Bayley-III),^50^ administered by an experienced developmental paediatrician (Andrew Chew). Cognitive and motor composite scores were evaluated [test mean (Sd) = 100(15)]. Autistic traits were assessed with the Quantitative Checklist for Autism in Toddlers (Q-CHAT), which is a parent-reported questionnaire designed to assess the risk of autism spectrum conditions (ASC) in toddlers and young children.^52^ Infants with confirmed genetic diagnoses and those born before 37 weeks gestational age (GA) were excluded from the statistical analyses exploring the relationship between GI *Z*-score and neurodevelopmental outcomes.

Neurodevelopmental outcome measures were analysed for 70 infants with CHD (5 infants died, 3 were unable to attend due to COVID-19 restrictions, 1 family emigrated, assessment was delayed for 1 child due to emergency surgery, 4 declined follow-up, 3 were lost to follow-up, 7 were excluded from analysis as they were born before 37 weeks GA age, 7 were excluded due to confirmed genetic abnormalities and 41 have not yet reached the 22-month developmental assessment time-point).

### Socioeconomic status

Index of multiple deprivation (IMD) was calculated using the mother’s post-code at birth for infants who attended the follow-up assessment. IMD is a composite measure of socioeconomic status in England encompassing factors such as income, employment, education, health and crime (https://imd-by-postcode.opendatacommunities.org/imd/2019; Accessed 21/11/2023). IMD was calculated from the 2019 data release and reported as scores and quintiles (most to least deprived). It was not possible to calculate IMD for two infants with CHD.

### Statistical analysis

A Shapiro–Wilk test was used to test normality. Two-tailed *t*-tests were performed to compare GI *Z*-scores between controls and infants with CHD for the whole brain and for specific regions, namely the frontal, occipital, parietal, temporal, cingulate and insular cortices for each of the two normative models. Further *t*-tests were performed, *post hoc*, to separately compare whole brain GI *Z*-scores between infants in each of the three CHD categories described above and the control cohort, and to compare whether there was any difference in outcome measures between male or female participants. Pearson’s correlation coefficient was calculated to determine the direction and strength of the relationship between brain GI *Z*-scores and CDO_2_. An ANCOVA was used to test for difference in CDO_2_ between cardiac groups, with PMA at scan as a covariate, and to test for differences in STBV between control and CHD infants, with PMA at scan, post-natal age at scan and sex as covariates. Partial Spearman’s Rank correlations were used to characterize the relationship between brain region GI *Z*-scores and neurodevelopmental outcome scores, with IMD rank included as a covariate for Bayley-III measures, and IMD rank and age at assessment as covariates for Q-CHAT scores. Benjamini–Hochberg False Discovery Rate was applied to correct for multiple comparisons (reported as *P*_FDR_). All statistical analyses were performed using statsmodels v0.13.2^53^ and Jupyter Notebook, python3.

## Results

### Participant characteristics

For infants with CHD, 142 participants met the inclusion criteria and were analysed. Of these, 60 (42%) had diagnoses categorized as ‘abnormal streaming of blood’, 43 (30%) had diagnoses categorized as ‘left-sided lesions’, and 39 (27%) had diagnoses categorized as ‘right-sided lesions’. For the dHCP cohort, data from 320 participants met the inclusion criteria. Demographics for all participants are summarized in Table 1, which includes information about individual diagnoses for infants with CHD. Seven infants with CHD also had genetic or syndromic diagnoses confirmed postnatally: five with 22q11.2 deletion (PA × 1, ToF × 2, Truncus arteriosus × 1, IAA × 1); one with CHARGE syndrome (ToF); and one with an SSR4 gene variant associated with X-linked disorder of glycosylation (CoA). After accounting for PMA at scan, post-natal age at scan and sex, STBV was significantly reduced in the CHD cohort compared to controls (*F* = 224, *P* < 0.0001) (Table 1).

**Table 1:** Participant demographics

	dCHP/controls (*N* = 320)	CHD			
		All (*N* = 142)	Abnormal streaming of blood (*N* = 60)	Left-sided cardiac lesions (*N* = 43)	Right-sided cardiac lesions (*N* = 39)
Gestational age at birth (weeks)	40.29 (39.29–41.00)	38.43 (37.71–38.86)	38.4 (38.0–38.9)	38.3 (37.6–38.9)	38.4 (37.6–38.9)
PMA at scan (weeks)	41.50 (40.43–43.00)	39.00 (38.46–39.71)	39.0 (38.6–39.6)	38.9 (38.3–39.6)	39.1 (38.6–40.1)
Time from birth to scan (days)	5 (2–15)	4 (2–7)	4 (2–7)	2 (1–5)	5 (2–7)
Sex	158 M:162 F	81 M:61 F	33 M:27 F	27 M:16 F	21 M: 18F
Birth-weight (kg)	3.426 ± 0.46	3.04 ± 0.55	3.10 (±0.47)	3.01 (±0.57)	3.00 (±0.61)
Head circumference (cm)	35.50 (34.48–36.50)	33.8 (32.8–35.0)	33.5 (33.0–34.5)	34.0 (33.0–35.0)	33.7 (32.5–35.0)
STBV (cm^3^)	338.68 (±42.7)	278.22 (± 33.14)	275.27 (±26.35)	279.25 (±40.00)	284.71 (±32.70)
CDO_2_ ml O_2_ min^−1^	-	1745 (±454)	1588 (±353)	1937 (±439)	1806 (±539)
Primary cardiac defect (*N*)			DORV = 4 TAPVD = 1 TGA = 50 TruA = 4 uAVSD = 1	cAS = 3 CoA = 28 HLHS = 8 IAA = 4	PA = 9 PS = 6 ToF = 19 TriA/D = 4 HRHS = 1

cAS, (critical) aortic stenosis; DORV, double outlet right ventricle; HRHS, hypoplastic right heart syndrome; TriA/D, tricuspid atresia or dysplasia; TruA, truncus arteriosus; uAVSD, unbalanced atrioventricular septal defect.

### GI is reduced in infants with CHD, proportional to reduced brain volume

**Figure 2:**
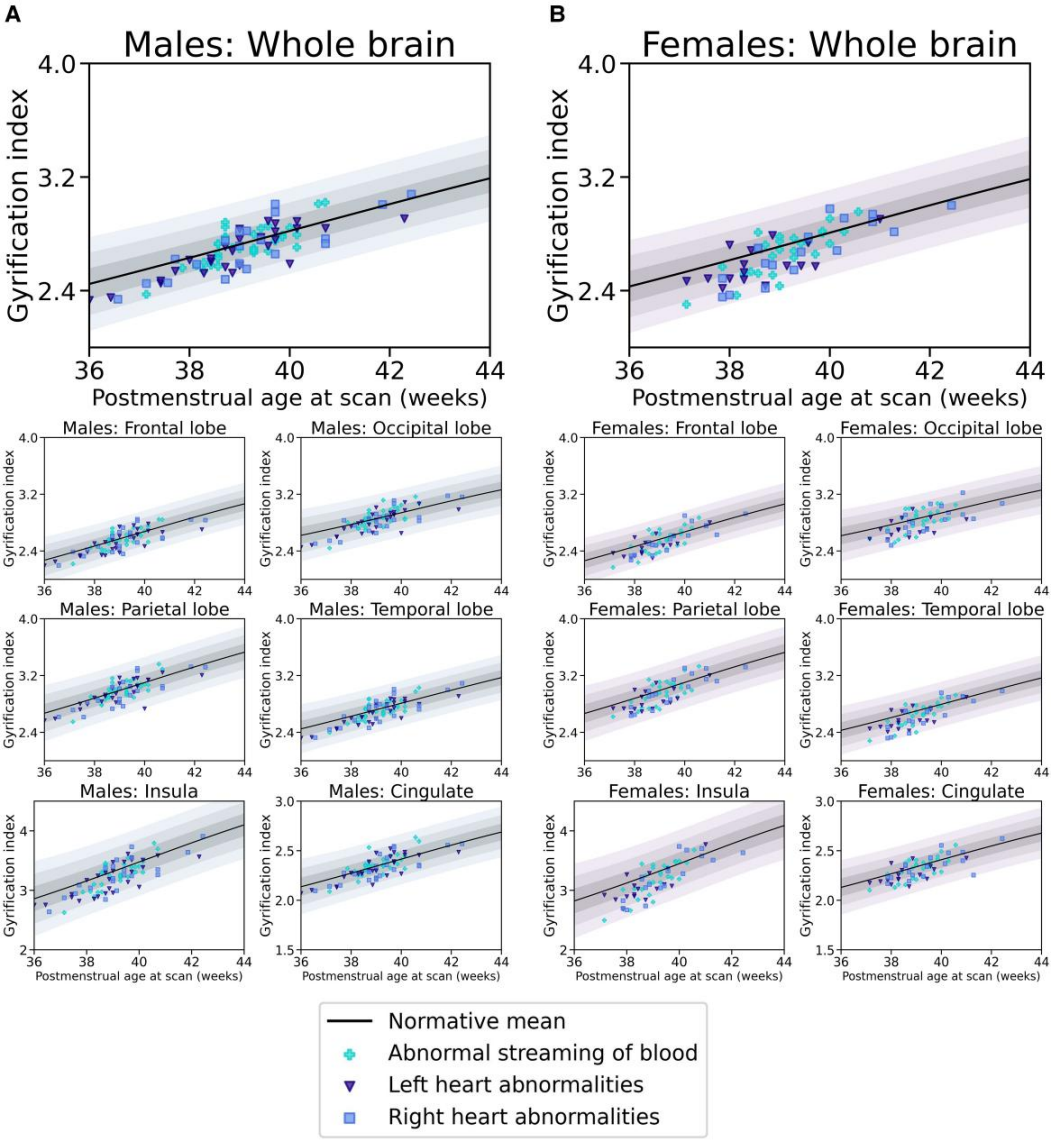
**Cortical GI measurements—normative Model 1.** Cortical GI measurements in 142 neonates with CHD overlaid on normative Model 1, accounting for PMA at scan, post-natal age at scan and sex. The normative mean derived from an independent population of 320 healthy control infants is shown as a black line. Shaded areas represent ±1, ± 2 and ±3 Sds from the normative model mean, separately for male (**A**) and female (**B**) infants. Individual data points for control neonates are not shown, for better visualisation.

**Figure 3:**
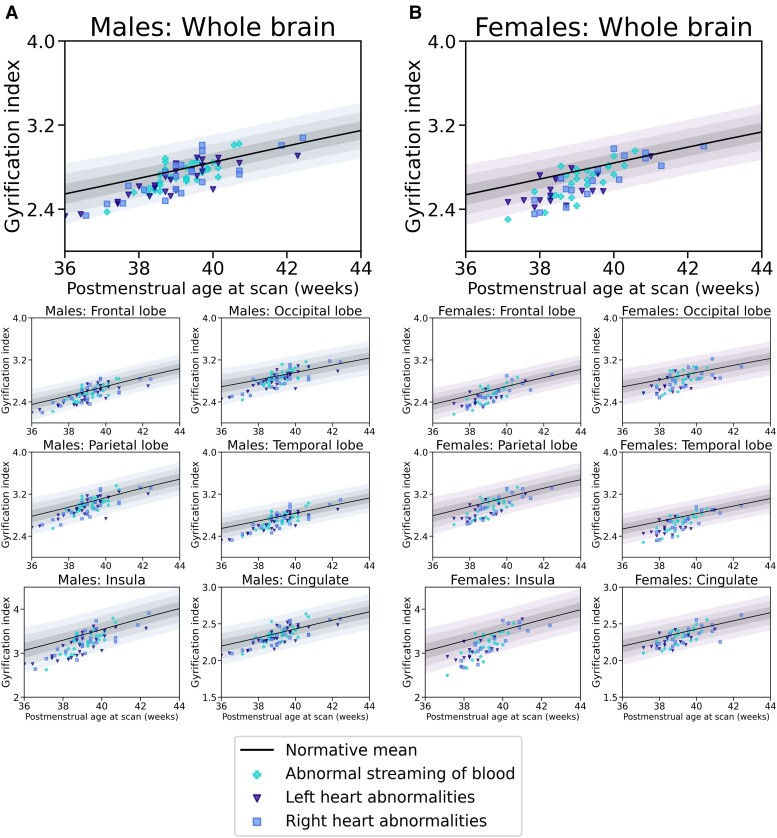
**Cortical GI measurements—normative Model 2.** Cortical GI measurements in 142 neonates with CHD overlaid on normative Model 2, accounting for PMA at scan, post-natal age at scan, sex and STBV. The normative mean derived from an independent population of 320 healthy control infants is shown as a black line. Shaded areas represent ±1, ± 2 and ±3 Sds from the normative model mean, separately for male (**A**) and female (**B**) infants. Individual data points for control neonates are not shown, for better visualisation.

**Figure 4:**
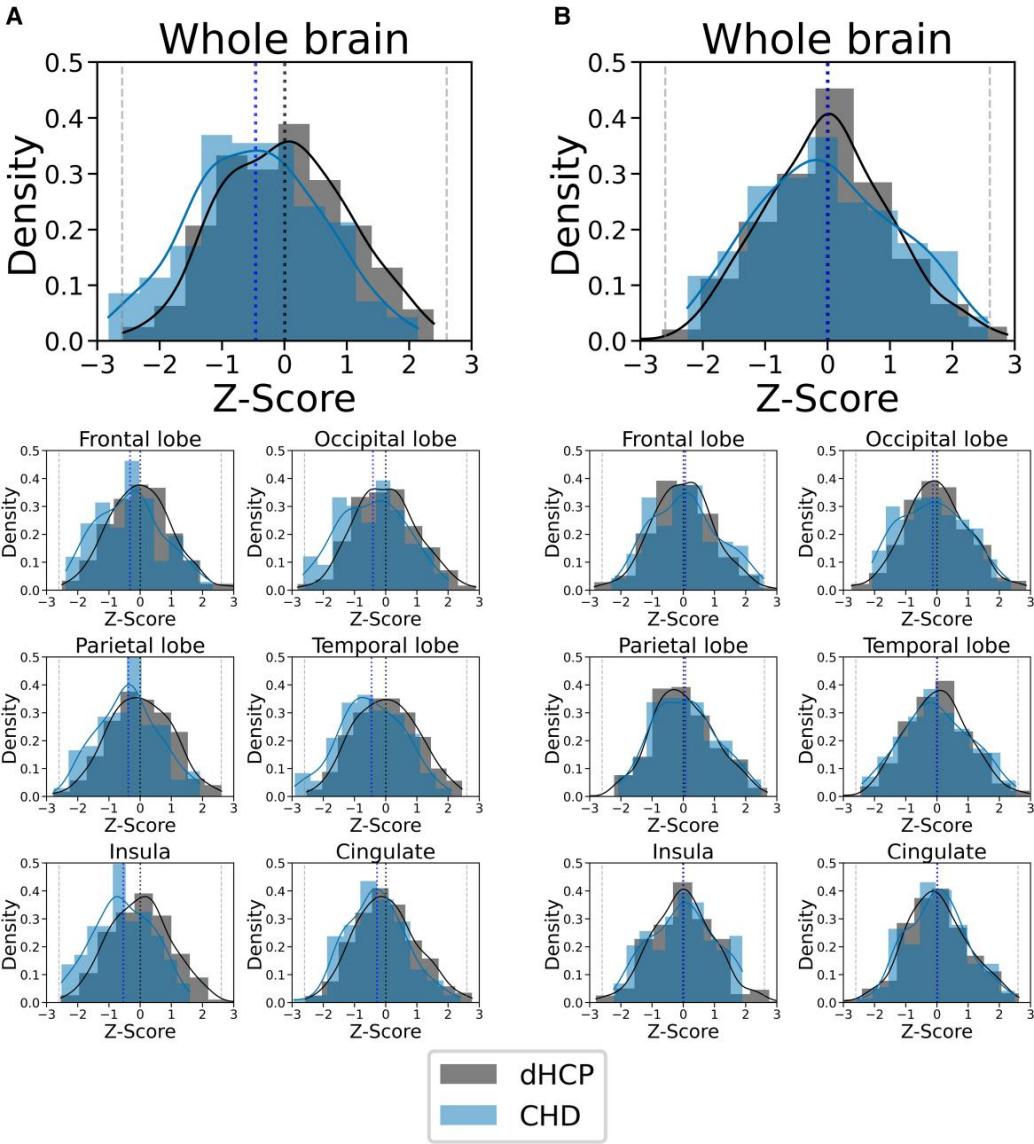
**GI *Z*-score histograms.** Histograms showing the distribution of GI *Z*-scores from normative Model 1 (**A**) and normative Model 2 (**B**) for the whole brain as well as the frontal, occipital, parietal, temporal, insular and cingulate cortices for both the healthy cohort (dHCP) (*N* = 320) and CHD infants (*N* = 142).

For normative Model 1, mean GI *Z*-scores were significantly lower for the whole brain, as well as the frontal, occipital, parietal, temporal, insular and cingulate cortices, in infants with CHD (all *P*_FDR_ < 0.005). When STBV was also included in the model (Model 2), there were no significant differences in GI *Z*-scores for the whole brain or any individual regions between infants with CHD and healthy controls (all *P*_FDR_ > 0.18). Mean absolute GI-values for both cohorts and GI *Z*-scores for the whole brain and each of the lobes analysed for infants with CHD for both models are shown in Table 2. Plots showing absolute GI measurements for all neonates with CHD overlaid on the normative mean and Sds, derived from the independent sample of 320 healthy infants, for both normative models are in Figs 2 and 3. Histograms showing the distribution of GI *Z*-scores for the whole brain and all brain regions analysed for both cohorts from both models are shown in Fig. 4.

**Table 2:** GI *Z*-score results for all infants with CHD, for the whole brain and individual brain regions

			Normative Model 1: Accounting for PMA at scan, post-natal age at scan and sex		Normative Model 2: Accounting for PMA at scan, post-natal age at scan, sex and STBV	
Region	GI (controls) (mean ± Sd)	GI (CHD) (mean ±SD)	Mean (±SD) Z-score	p_FDR_	Mean (±SD) Z-score	p_FDR_
Whole brain	2.96 (±0.18)	2.68 (±0.17)	−0.47 (±1.07)	**<** **0**.**001**	−0.0030 (±1.11)	0.92
Frontal lobe	2.82 (±0.18)	2.54 (±0.17)	−0.32 (±1.11)	**0**.**002**	0.0043 (±0.99)	0.60
Occipital lobe	3.06 (±0.19)	2.83 (±0.17)	−0.41 (±1.11)	**<** **0**.**001**	−0.14 (±1.16)	0.18
Parietal lobe	3.27 (±0.20)	2.96 (±0.19)	−0.38 (±0.96)	**<** **0**.**001**	0.0047 (±1.01)	0.54
Temporal lobe	2.94 (±0.18)	2.67 (±0.17)	−0.46 (±1.05)	**<** **0**.**001**	−0.0042 (±1.10)	0.92
Insula	3.70 (±0.32)	3.21 (±0.29)	−0.54 (±0.90)	**<** **0**.**001**	−0.013 (±1.02)	0.85
Cingulate	2.52 (±0.14)	2.34 (±0.12)	−0.28 (±0.90)	**0**.**005**	0.010 (±0.96)	0.96

*P*_FDR_-values are from a *t*-test comparing the mean *Z*-score for this cohort with the mean *Z*-scores derived from a cohort of 320 healthy infants. Results in bold are significant (*P*_FDR_ < 0.05). Results are shown for both normative models: One accounting for the infant’s PMA at scan, post-natal age at scan (i.e. the number of days since birth), and sex, and a second which accounted for STBV in addition to PMA at scan, post-natal age at scan and sex.

**Figure 5:**
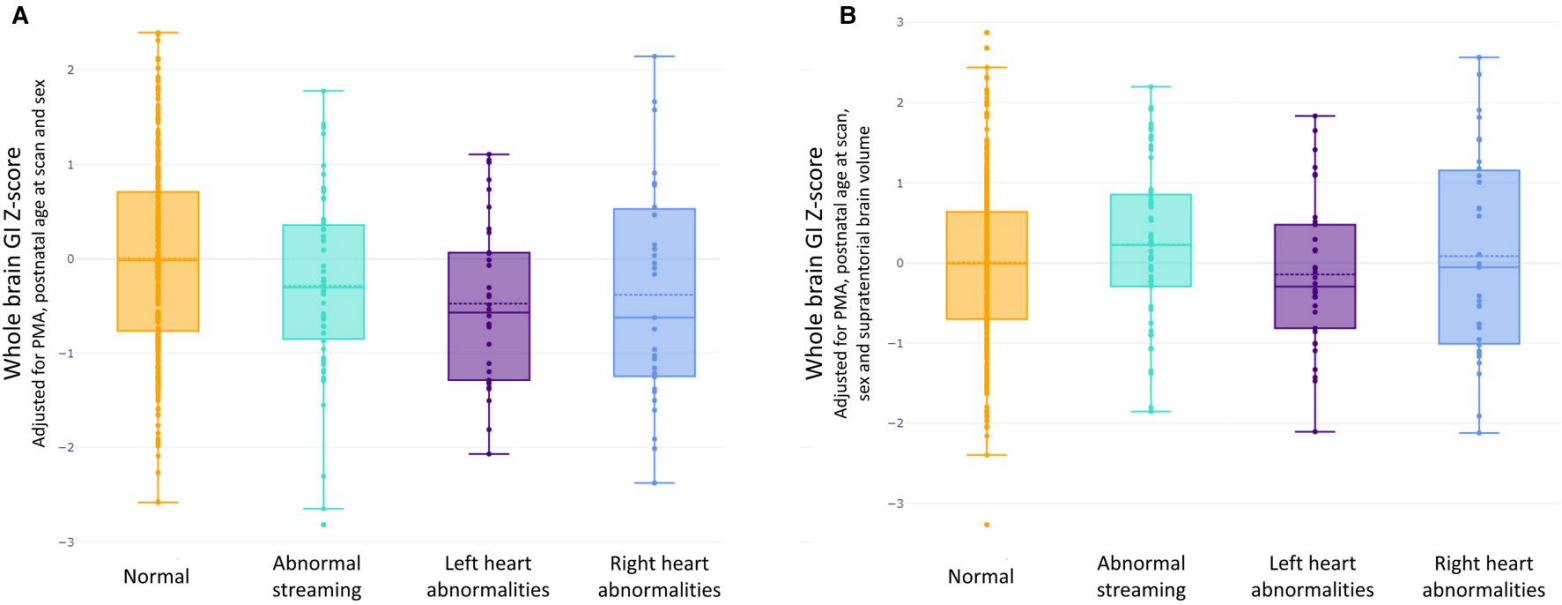
**Whole brain GI *Z*-scores.** Whole brain GI *Z*-scores for all healthy control infants (orange) and three categories of infants with CHD (abnormal streaming, *N* = 60, left-sided cardiac lesions, *N* = 43, right-sided cardiac lesions, *N* = 39), based on the haemodynamic impact of the underlying cardiac diagnosis. Two-tailed *t*-tests were used to show that mean whole-brain GI *Z*-scores derived from a normative model accounting for PMA, post-natal age at scan and sex are significantly reduced in all three groups when compared to control infants (*N* = 320) (**A**) (abnormal streaming = − 0.29 (*t* = 2.01, *P*_FDR_ = 0.045); left-sided lesions = − 0.67 (*t* = 2.71, *P*_FDR_ = 0.021); right-sided lesions = − 0.63 (*t* = 2.04, *P*_FDR_ = 0.045)), but that there were no significant reductions in mean whole-brain GI *Z*-scores derived from a normative model accounting for PMA, post-natal age at scan, sex and STBV in any of the three cardiac groups when compared to control infants (*N* = 320) (**B**) (abnormal streaming = 0.23 (*t* = −1.54, *P*_FDR_ = 0.37); left-sided lesions = − 0.14 (*t* = 0.80, *P*_FDR_ = 0.63); right-sided lesions = 0.090 (*t* = − 0.43, *P*_FDR_ = 0.67)). In both plots, solid lines represent the median value for each group; dashed lines represent the mean.

When comparing CHD groups to the normative mean, whole-brain GI *Z*-scores derived from Model 1 were significantly reduced in all three groups when compared to controls [abnormal streaming = −0.29 (*t* = 2.01, *P*_FDR_ = 0.045); left-sided lesions = −0.67 (*t* = 2.71, *P*_FDR_ = 0.021); right-sided lesions = −0.63 (*t* = 2.04, *P*_FDR_ = 0.045)]. However, there were no significant reductions in whole-brain GI *Z*-scores derived from Model 2 in any of the three cardiac groups when compared to controls [abnormal streaming = 0.23 (*t* = −1.54, *P*_FDR_ = 0.37); left-sided lesions = −0.14 (*t* = 0.80, *P*_FDR_ = 0.63); right-sided lesions = 0.090 (*t* = −0.43, *P*_FDR_ = 0.67)] (Fig. 5).

### GI is associated with CDO_2_ in infants with CHD, but not when brain volume is accounted for

**Figure 6:**
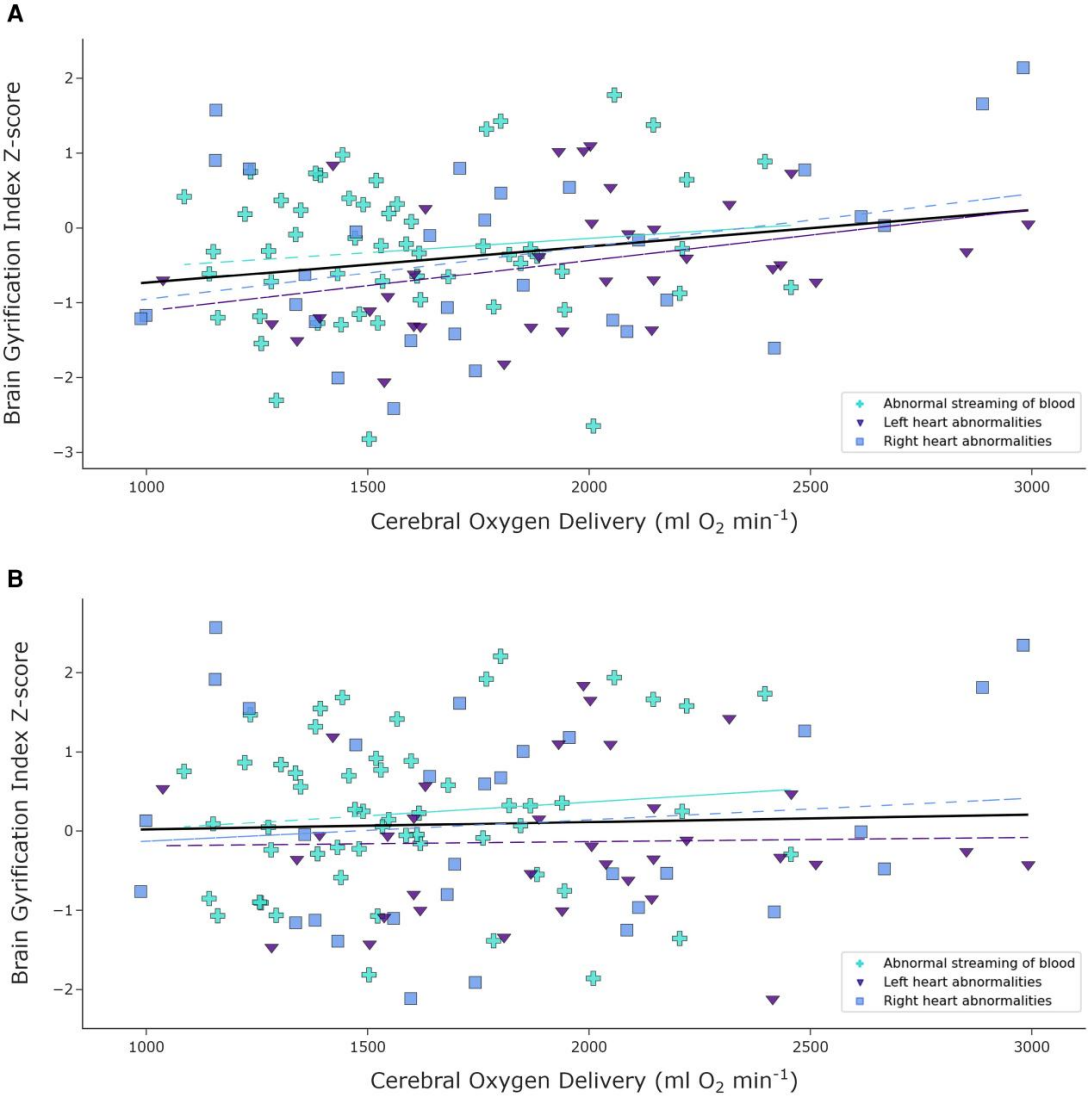
**CDO_2_ and whole brain GI.** CDO_2_ demonstrates a significant positive correlation with whole brain GI *Z*-scores derived from normative Model 1, which accounts for PMA, post-natal age at scan and sex (**A**) (partial Spearman’s rank correlation *R*^2^ = 0.05, *P* = 0.015), but not from normative Model 2, which also accounts for STBV (**B**) (partial Spearman’s rank correlation *R*^2^ = 0.002, *P* = 0.66) in 120 neonates with CHD, after accounting for PMA at scan, post-natal age at scan and neonatal sex.

CDO_2_ [mean (±Sd) 1745 (±454) ml O_2_ min^−1^] was significantly positively correlated with whole brain GI *Z*-scores derived from Model 1 (*R*^2^ = 0.05, *P* = 0.015) (Fig. 6). Here, there was a significant difference in CDO_2_ between cardiac groups (*P* = 0.0011) after accounting for PMA at scan, with mean CDO_2_ being lowest in the group with abnormal streaming of blood (Table 1). However, there was no significant correlation between CDO_2_ and whole brain GI *Z*-scores derived from Model 2 (*R*^2^ = 0.002, *P* = 0.66) (Fig. 6).

### Whole brain gyrification is not associated with neurodevelopmental outcomes

Cognitive composite outcome scores were available for 70 infants. Motor composite outcome scores were available for 69; Q-CHAT total scores were available for 66. Demographics at follow-up, outcome scores and IMD scores for all infants with CHD and each of the three CHD groups are shown in Table 3. There was no significant difference in cognitive composite scores, motor composite scores or Q-CHAT scores for male and female participants (cognitive composite score, *T* = −0.45, *P* = 0.66; Motor composite score, *T* = −1.0, *P* = 0.32; Q-CHAT score, *T* = −0.95, *P* = 0.34).

**Table 3:** Demographics at follow-up for infants with CHD

	All (*N* = 70)	Abnormal streaming of blood (*N* = 29)	Left-sided lesions (*N* = 24)	Right-sided lesions (*N* = 17)
Age at follow-up corrected for gestational age at birth in months, median [interquartile range (IQR)]	22.26 (22.03–23.47)	22.3 (22.1–24.1)	22.2 (22.1–22.8)	22.3 (22.0–22.5)
Cognitive composite score, mean (±Sd)	93 (±12)	93 (±11)	94 (±14)	94 (±9)
Motor composite score, mean (±Sd)	94 (±12)	96 (±10)	96 (±14)	93 (±10)
Q-CHAT score, mean (±Sd)	29 (±8)	30 (±8)	28 (±10)	31 (±6)
IMD score, median (IQR)	18.2 (12.3–31.8)	16 (11–31)	14 (11–25)	27 (14–37)
IMD quintiles	Number (%)			
First (most deprived)	14 (21)	5 (7)	2 (3)	7 (10)
Second	15 (22)	6 (9)	6 (9)	3 (4)
Third	12 (18)	5 (7)	4 (6)	3 (4)
Fourth	13 (19)	8 (11)	7 (10)	3 (4)
Fifth (least deprived)	14 (21)	5 (7)	5 (7)	1 (1)

When using GI *Z*-scores from either of the two normative models, there was no association between whole brain GI *Z*-score and cognitive composite scores (Model 1: *⍴* = 0.047, *P* = 0.70; Model 2: *⍴* = 0.010, *P* = 0.93) or motor composite scores (Model 1: *⍴* = 0.13, *P* = 0.30; Model 2: *⍴* = 0.046, *P* = 0.72), after accounting for IMD. There was no association between whole brain GI *Z*-score derived from either model and Q-CHAT total scores, after accounting for IMD and age at assessment (Model 1: *⍴* = 0.14, *P* = 0.26; Model 2: *⍴* = −0.17, *P* = 0.17).

## Discussion

This study used normative modelling to explore individualized measures of cortical gyrification in 142 neonates with critical or severe CHD prior to cardiac surgery. We observed a large degree of heterogeneity in individualized measures of cortical gyrification. When accounting for PMA at scan, post-natal age at scan and sex, whole brain and lobar gyrification was reduced in infants with CHD in the preoperative neonatal period, with whole brain GI *Z*-scores nearly half a standard deviation lower than when compared to typical development. However, when also accounting for STBV, there was no significant difference in whole brain or lobar gyrification in these infants with CHD. This finding suggests that whilst brain folding is reduced in CHD, it is primarily driven by the fact that brain size is reduced in CHD.

Our finding of reduced GI in infants with CHD is consistent with previous neonatal MRI studies,^15,28,29^ and complementary to other work showing that cortical folding is delayed in foetuses with HLHS.^26^ We used a previously published categorisation approach for the CHD cohort, grouping infants according to the haemodynamic impact of their underlying cardiac diagnosis. *Post hoc* analyses revealed that GI *Z*-scores derived from Model 1 were significantly reduced compared to controls in all three CHD groups. These findings support existing work showing that cortical gyrification is reduced in infants with severe CHD, but which did not study infants with right-sided cardiac lesions,^29^ or which only studied a small cohort of those with single-ventricle physiology,^28^ and, crucially, did not account for brain volumes when assessing cortical folding.

Animal studies have shown that cortical development is vulnerable to *in utero* changes in cerebral oxygenation. Cerebral ischaemia impairs cortical volumetric growth and results in altered cortical microstructure in sheep fetuses,^54^ and cortical gyrification is significantly reduced even after brief periods of cerebral hypoxia.^55^ Similarly, in a porcine model of CHD, cerebral hypoxia resulted in decreased neuronal proliferation, migration and overall volume of the cortex, and was also associated with a reduction in GI.^56^ Post-mortem histological studies of the brain in human infants with CHD have also revealed immature astrocytic processes, suggesting that CHD depletes progenitor cell pools that are important for normal cortical growth,^56^ and that the cortex appears immature in HLHS.^25^ In the context of these studies, the reduced GI we report may reflect impaired cortical development resulting from cerebral hypoxia *in utero* and postnatally, which occurs due to altered cerebrovascular haemodynamics in CHD.^15,57-59^ However, given the finding that GI is not reduced in CHD when brain volume is accounted for, the primary cerebral phenotype in CHD may actually be smaller brain volumes, with proportionate reductions in cortical folding, rather than specifically disrupted folding mechanisms.

In addition to the reduction in whole brain GI *Z*-scores from Model 1 seen in the CHD cohort, regional analyses revealed that the insular and the temporal lobe cortices showed the greatest reductions in folding when compared to controls. A widened, or more ‘open’, operculum has been reported in several neonatal MRI studies in the CHD population,^28,60-62^ which is likely to be a manifestation of reduced cortical folding in this area. The technique used in this study may provide a quantitative, individualized measure of cortical folding in the opercular region with which to assess this.

### Cortical gyrification is associated with CDO_2_, but not when brain volume is accounted for

Similar to previous work in a smaller CHD cohort,^15^ when accounting for PMA at scan, post-natal age at scan and infant sex, the degree of cortical folding was significantly associated with CDO_2_. However, when also accounting for STBV, there was no significant correlation between CDO_2_ and GI *Z*-scores. Furthermore, when accounting for PMA at scan, post-natal age at scan and infant sex, CDO_2_ only accounted for ∼5% of the variance in whole brain GI. Previous work performed in a sub-sample of the same cohort showed that CDO_2_ explained ∼18% of the variance in cGM volumes in infants with CHD.^31^ These findings suggest that CDO_2_ in the early neonatal period plays a more important role in the biological processes underlying volumetric brain growth than cortical folding and supporting the hypothesis that differences in GI between infants with CHD and controls are explained by STBV. This implies that strategies to improve cerebral oxygenation may help correct the altered trajectory of impaired brain growth observed in this population.

It is plausible that the biological processes behind cortical volume growth and cortical gyrification have different sensitivities to cerebral oxygen levels. Early cortical volumetric growth is primarily determined by neuronal and glial cell proliferation, which animal studies have shown to be sensitive to hypoxaemia.^63-66^ In contrast, gyrification is influenced by both mechanical factors and cortical maturation processes,^67^ including axonal tension in cortico-cortical projections.^68,69^ Folding of the primary cortical sulci is also under strong spatial-temporal genetic control,^70-72^ with post-natal changes in cortical development occurring as a result of genetically programmed developmental processes.^73^ Furthermore, whilst cortical volumetric expansion and folding are highly temporally associated, the periods during which they take place do not completely overlap, with cortical differentiation and volumetric growth occurring before any sulci emerge.^9,74^

### Whole brain gyrification is not associated with neurodevelopmental outcomes

To our knowledge, this is the first study exploring the potential link between cortical gyrification in the preoperative neonatal period and neurodevelopmental outcomes in early childhood in individuals with CHD. We did not identify any associations between whole brain GI *Z*-scores derived from either normative model or measures of motor function, cognitive ability, or ASC traits. The relationship between cGM development trajectory in infants with CHD and subsequent neurodevelopmental outcome is not clear. We have shown previously that reductions in cortical and deep grey matter volumes in infants with CHD were significantly associated with poorer cognitive abilities at 22 months,^31^ and smaller cGM volumes were associated with lower Bayley-III fine motor scores at 9 months of age in one study involving a diagnostically heterogeneous cohort of 51 infants with CHD.^75^ However, another study looking at post-operative cGM volumes in 30 infants with CHD did not identify any association with full-scale IQ at 6 years of age.^76^

In this study, cortical gyrification was measured postnatally but prior to any cardiac surgery. An important consideration is that additional surgical and clinical factors may influence later brain development and neurodevelopmental outcomes, such as the number of surgical interventions, nature and duration of cardiac bypass, and post-surgical hospitalisation time.^77-81^ Social and environmental factors are also important influences on neurodevelopmental outcomes in CHD.^82-84^ To partially address this, we included IMD as a covariate in the analyses between whole-brain GI *Z*-score and neurodevelopmental outcomes as a measure of socioeconomic status. Clearly, the relationship between measures of cortical development in neonates with CHD and subsequent neurodevelopmental outcomes is complex, and future studies, with more informative outcomes in later childhood and beyond, are required.

### Limitations

Whilst the CHD cohort included in this study is large and diagnostically heterogeneous, it is limited by the relatively small numbers of infants with HLHS. Additionally, neurodevelopmental assessments of cognitive and behavioural outcome were performed at 22 months of age and interpretation is limited by the incomplete follow-up of all participants. Where they exist, any associations may not become evident until a later age.^85,86^

## Conclusion

Individualized measures of cortical gyrification, in the form of GI *Z*-scores, are significantly reduced preoperatively in a large cohort of infants with CHD when accounting for PMA at scan, post-natal age at scan and sex but, crucially, GI *Z*-scores are not reduced in the CHD group when STBV is also accounted for. This finding suggests that whilst brain folding is reduced in CHD this reduction is proportional to reductions in brain volume, rather than being specifically related to disrupted folding mechanisms. Given this, it is important that future studies assessing cortical folding in infants with CHD account for brain volume. The insular and temporal lobe cortices display the greatest deviation in GI from the normal trajectory. Reductions in whole-brain cortical folding are associated with decreased CDO_2_ in the early neonatal period, but again, not when STBV is accounted for. CDO_2_ is therefore likely to play a more important role in the biological processes underlying volumetric brain growth than cortical folding. Impaired brain growth in CHD with proportional reductions in folding are likely to result from *in utero* and post-natal cerebral hypoxia associated with CHD. However, cortical folding in the preoperative period was not associated with neurodevelopmental outcomes at a median age of 22 months in CHD.

## Data Availability

The data that support the findings of this study are available from the corresponding author, upon reasonable request. The GPR model and code used in this study is freely available on GitHub (https://github.com/ralidimitrova/GPR_NeoVols).
